# Effects of local phenytoin on seroma formation after mastectomy and Axillary lymph node dissection: an experimental study on mice

**DOI:** 10.1186/1471-2482-12-25

**Published:** 2012-12-19

**Authors:** Mehmet Eser, Fırat Tutal, Metin Kement, Selcuk Goktas, Levent Kaptanoglu, Mehmet Gökceimam, Melin Ozgun Gecer, Huseyin Uzun

**Affiliations:** 1 Department of General Surgery, Kartal Training and Research Hospital, Kartal, Istanbul, Turkey; 2 Department of Pathology, Kartal Training and Research Hospital, Kartal, Istanbul, Turkey

**Keywords:** Mastectomy, Lymph node dissection, Breast cancer

## Abstract

**Background:**

Seroma formation is the most common complication after breast cancer surgery. It is an important complication since it prolongs therapy duration, increases cost, and negatively affects patient psychology. Currently, there is no widely accepted method that prevents seroma formation. We tried to investigate impact of local phenytoin application on seroma formation following an experimental mastectomy model created in rats.

**Methods:**

Two groups including eight rats in each were randomized. Saline injection was applied in the first group, whereas 1% phenytoin was locally used in the second group. Ten days after the surgery, seroma formation and wound-healing processes were evaluated using histopathological and biochemical examinations.

**Results:**

Phenytoin significantly decreased seroma formation. Fibrosis was significantly increased and angiogenesis was significantly reduced in the phenytoin group (P < 0.05). Increased levels of macrophage and lymphocyte infiltration was detected in the control group (P < 0.05). No difference was detected between the groups in terms of necrosis, edema, congestion, and PNL (Polymorphonuclear leucocyte) and fibroblast infiltration.

**Conclusions:**

Seroma formation-reducing effect of phenytoin might have occurred over its anti-inflammatory, anti-angiogenetic, and fibrosis augmenting effects.

## Background

Seroma formation isone of the most common complications after breast cancer surgery. It has been reported that seroma occurs by 5-52% after modified radical mastectomy (MRM) procedure and by 7.1-14% after sentinel lymph node biopsy (SLNB) [1-5].

Seroma formation usually disappears following surgery within a few weeks. But sometimes it may persist for a few months. Prolonged therapy duration and requirement for multiple aspirations may cause trouble for patients, and more importantly it can delay adjuvant therapy and may cause increasement of treatment costs.

We believe flaps are lightly attached to chest wall, and additionaly perioperative dissection may harm lymphatic and capillary vessels. Large dead space, auxillary effects of muscles to venous return may be interrupted during surgery. Finally shape of the axilla and chest wall, local inflammatory mediators and all factors mentioned above are considered responsible for seroma formation [2]. Seroma formation is seen more likely in overweight patients, patients that underwent modified radical mastectomy as opposed to simple mastectomy, and in those with increased amount of drainage within the first three days of surgery [3]. Rather than treatment of seroma, due to ineffective therapy methods, surgeons directed their interests to prevent formation. One of the best methods in preventing seroma formation is to get rid of dead-space. Meticulous dissection and application of various medical agents may accelerate wound healing and fibrosis. Despite certain success rates obtained by improvement of surgical techniques and usage of fibrin glue, these therapy modalities have failed to become routine clinical applications [6-8]. It has been reported that phenytoin, which is an antiepileptic agent, has also antibacterial effect, which can accelerate wound healing and reduce pain [9,10]. The present study aimed to investigate the effect of local phenytoin application on seroma formation in an experimental mastectomy and ALND model in rats.

## Methods

**Figure 1:**
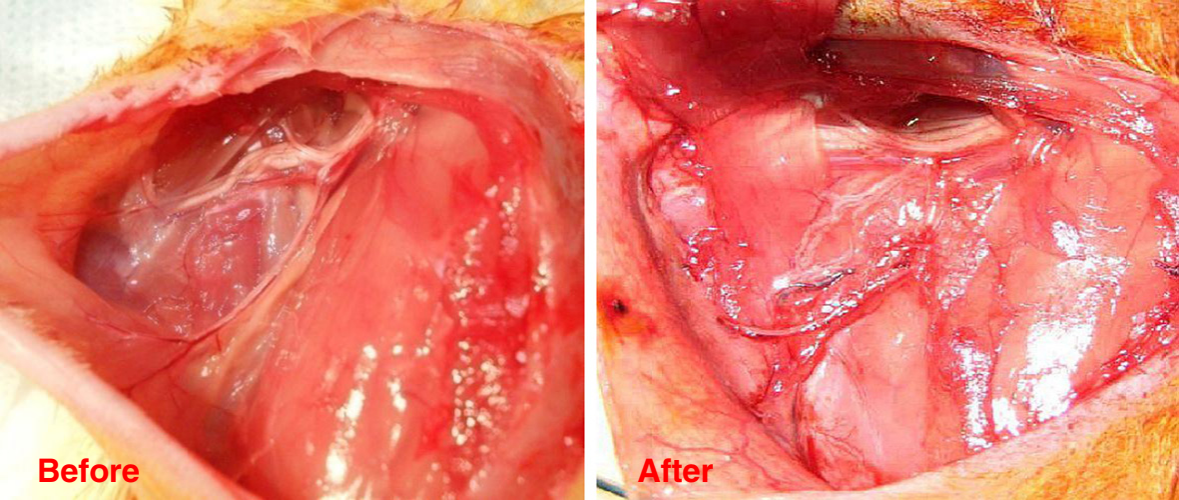
Axillary adipose cellular tissue before and after axillary dissection.

Sixteen female Wistar Albino rats (mean weight 220 g) were utilized in the present study. Approval of Istanbul Yeditepe University of Medical Faculty, Ethical Committee on Experimental Animal Research was obtained (2011–186). Animals were obtained from the Experimental Animals Laboratory of Istanbul Yeditepe University of Medical Faculty. All rats were fed with standard laboratory food and water [rodent chow ad libitum] and monitored in an isolated place, which had controlled heat (22 ± 2°C) and was illuminated as 12-hour night/day cycle. Surgeries were performed in Istanbul Yeditepe University of Medical Faculty Experimental Animals Laboratory under non-sterile but clean conditions. Preoperative prophylactic antibiotics were not used to avoid drug interaction. Animals were weighed prior to the surgery. A sterile form of 1% phenytoin in normal saline was prepared. Exclusion criteria were determined as follows: flap necrosis, wound dehiscence, infection, and death. Ketamine (Ketalar®, Parke Davis and Co. Inc. USA, 50 mg/kg) and xylazine (Rompun®, Bayer, Germany 5 mg/kg) were used for anesthesia. An incision beginning from the sternal notch and extending to the xiphoid was made in all rats in accordance with the method defined by Harada et al. [7]. Flaps were prepared by detaching the skin and the subdermal tissues from the thoracic wall. Major pectoral muscle was excised from the thoracic wall. Axillary artery and vein were preserved. Axillary adipose tissues were excised; unilateral mastectomy and ALND were performed (Figure 1). Neither cautery nor chemical substance was used for hemostasis. Incision was closed using 4/0 polyglactin and 4/0 silk. Rats were divided into two groups including eight rats in each.

In the control group; normal saline, the volume of which corresponded to the appropriate phenytoin dose that has been adjusted according to the weight of the rats, was applied under the flaps, immediately following abdominal closure. In the phenytoin group; 1% phenytoin solution, which had been prepared before, was applied under the flaps at a dose of 4 mg/kg.

Rats were monitored for 10 days after the surgery. Viability of the flaps, movement of the extremities, wound healing, wound infection, necrosis seroma formation were evaluated. On the 10^th^ day postoperatively seroma was aspirated under anesthesia with needle and recorded. Relaparotomies were done and remnant seroma was aspirated and measured. Tissue samples from the thoracic wall and axilla were also obtained for histopathological examination. They were fixed with 10% formalin solution. After these procedures, rats were sacrificed by decapitation with a guillotine.

Histopathological examination: After tissue samples were embedded in paraffin blocks, 5 μm sections were obtained. Preparations were stained with hematoxylin and eosin (HE) and with Masson's Trichrome stain to evaluate fibrosis better. Angiogenesis, edema, necrosis, congestion and augmentation of fibrous tissue were examined quantitatively under the light microscope and were scored as follows: Grade 1: No or mild alteration, Grade 2: Moderate alterations, Grade 3: Increased alterations, Grade 4: Intensive alterations. Polymorphonuclear leukocyte (PNL), fibroblast, lymphocyte and macrophage infiltrations were scored semi-quantitatively as follows: Grade 1: no or minimal cell infiltration, Grade 2: moderate infiltration, Grade 3: increased infiltration, Grade 4: intensive infiltration.

Statistical analyses were done using ‘SPSS Data Editor for Windows version 17.0’ program. Seroma volume and histopathological parameters were evaluated by Mann–Whitney *U* test. A p value of <0.05 was considered significant.

## Results

None of the rats developed wound-related problem [dehiscence, infection, macroscopic flap necrosis, etc.] or a problem that would cause their exclusion from the study. All animals survived. Temporarily movement limitations was observed in the upper extremity at the same side where mastectomies were done within the postoperative 3 days.

The mean seroma volume was 1.2 ± 0.11 ml in the control group, whereas it was 0.51 ± 0.08 ml in the phenytoin group. This difference was significantly different (p = 0.001).

**Figure 2:**
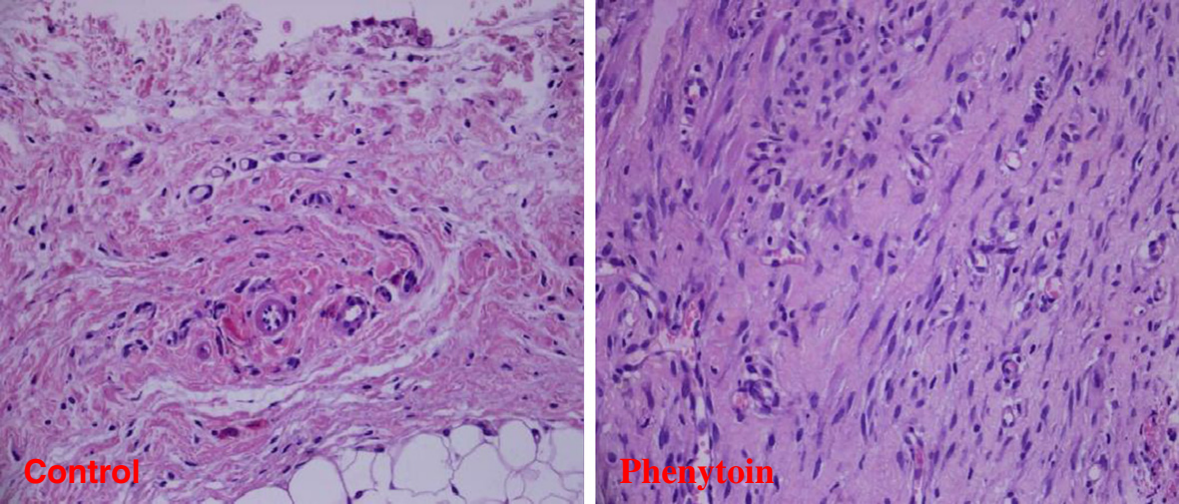
There was also significant microscopic difference between the control and phenytoin groups in terms of fibrous tissue enhancement.

Histopathological findings: Fibrosis was found significantly higher (Figure 2) and angiogenesis was found significantly lower in the phenytoin group as compared to the control group (P < 0.05). However, macrophage and lymphocyte infiltrations were significantly higher in the control group (P < 0.05). There was no difference between the groups in terms of necrosis, edema, congestion, or PNL and fibroblast infiltration (Table 1).

**Table 1:** Semi-quantitative values of histopathological findings

	**Phenytoin**	**Control**	**P value**
Vascularity [angiogenesis]	2.75 ± 0.46	3.38 ± 0.52	0.029
Necrosis	1.25 ± 0.70	1.00 ± 0.00	0.317
Congestion	2.50 ± 0.54	2.13 ± 0.35	0.124
PNL	1.50 ± 0.54	1.38 ± 0.52	0.629
Lymphocyte	1.63 ± 0.52	2.13 ± 0.35	0.044
Edema	1.63 ± 0.52	2.25 ± 0.71	0.069
Fibrosis	3.25 ± 0.71	2.25 ± 0.71	0.019
Fibroblast	3.00 ± 0.76	2.88 ± 0.64	0.725
Macrophage	1.25 ± 0.46	2.13 ± 0.64	0.011

## Discussion and conclusion

Seroma is a serious fluid collection in a surgical cavity that is clinically evident. Seroma formation is the most common complication after mastectomy and occurs at a rate of 5-52% (mean 30%) under the skin flap or in the dead-space in the axilla [1-3,6,11].

Pathogenesis of seroma formation is unclear. Analysis of the seroma that occurred after mastectomy and axillary curettage revealed an exudative fluid including cellular components of acute inflammation [12]. Dead-space after mastectomy is initially filled with a fluid consisting of blood and lymphatic leak. During inflammatory process of surgical trauma, polymorphonuclear leukocytes (PNL) and macrophages migrate to this area due to the effects of histamine, prostaglandin and adenosine in particular, and the vascular ends, which have been closed due to vasoconstriction, open and contribute to the fluid flow. In addition, concentration of soluble particles in the fluid changes with phagocyte infiltration. Osmotic pressure gradient occurs when particle concentration is higher in the seroma than surrounding tissue. Accordingly, it is thought that fluid flow to this area continues until particle concentrations become equal, and thus, seroma persists [13,14].

Many factors are thought to be associated with seroma formation after mastectomy and axilla dissection. Kuroi et al. investigated evidence level of these factors in a review consisted of 51 randomized, 7 prospective and 7 retrospective studies. They reported that seroma formation was not associated with previous biopsy, type of drainage (closed suction versus static drainage), duration of drainage, number of drains, intensity of negative suction pressure, removal of drains on the fifth postoperative day or when daily drainage volume fell to minimal, immobilization of shoulder, lymph node status or lymph node positivity, number of removed lymph nodes, hormone receptor status, and stage. They also reported moderate level of evidences concerning increased risk for seroma formation in overweight patients, as well as in the patients that underwent modified radical mastectomy as compared to simple mastectomy and in the patients that had higher amount of drainage within the first three days of surgery [3]. We preferred Harada et al. method in our experimental study. This is well documented and frequently used model. Pectoral muscle is excised in this model. There are contraversial thoughts about cautery application, due to its hegative impact on seroma formation. Because of that we tried to abstain from cautery usage. Cautery application in humans differs according to surgeons choice. Some prefers cautery only for the whole procedure. We tried to standardise our experiment and did not use cautery. All the surgeries could have done with the help of coagulation.

Breast surgeries in humans generally needs prophylactic antibiotics. Since these operations are considered as clean, routine application of antibiotics is not mandatory. Also in our experimental study we could have seen infection. Due to its diminished possibility we preferred to abstain from antibiotic usage. There was no infection in animals.

Numerous methods including surgical techniques [6,15,16], pressure dressings [17], immobilization [18,19], closed drain suction devices [20,21], sclerosing agents [6], and various types of tissue adhesives have been investigated in terms of their ability to minimize seroma formation. Nevertheless, these methods are not always effective and may contribute additional morbidity for patients. Although many studies have shown that surgical closure of dead-space is an effective method, it has not been widely accepted since it prolongs surgery by 10 to 20 minutes [22].

Many studies have shown favorable effects of phenytoin on wound healing. It was reported in those studies that phenytoin reduced inflammation, pain, bacterial contamination and exudate in the wound but augmented fibroblastic proliferation, granulation tissue formation, neo-vascularization and collagenization [9,10,23-25]. Depending on favorable effects of phenytoin on wound healing, particularly exudate-reducing effect in the wound, we decided to conduct the present experimental study thinking that phenytoin might have seroma-reducing effect as well. In the present study, the decrease in seroma volume of the phenytoin group was found statistically significant (P = 0.001). Histopathological examination revealed significantly lower angiogenesis and lymphocyte and macrophage infiltration but significantly higher fibrosis. The mechanism of seroma preventing effect of phenytoin is unclear. In the present study, we observed that phenytoin had anti-inflammatory and anti-angiogenic but fibrosis enhancing effects. Seroma-reducing effect of phenytoin might occur from the above-mentioned effects. Excess fluid collection in the surgical trauma area may result from excess wound inflammation in this area. This hypothesis seems to be corroborated by the study of Watt-Boolsen et al., which showed that seroma is a kind of inflammatory exudate and that seroma formation reflects prolongation and severity of inflammatory phase of wound healing [26]. One of the significant findings of the present study is reduced angiogenesis in the phenytoin group. It was shown that vasodilatation is remarkable in the inflammatory phase of wound healing and plays an important role in extravasation of fluid [27,28]. Prevention of augmentation of angiogenesis and vasodilatation might reduce seroma formation due to decreased fluid flow from capillary bed to dissection area. In their rat model of mastectomy, Kocdor et al. reported that 5 FU might have reduced seroma due to its anti-inflammatory and anti-angiogenetic effects [29]. The other significant finding of the present study is incerasement of fibrosis by phenytoin. Augmented fibrosis in the surgical area might be important in terms of adhesion of flaps to the surgical area and filling of dead-space with fibrosis. In conclusion, local phenytoin application reduces seroma formation in the experimental rat model of mastectomy. Mechanism of seroma-reducing effect of phenytoin and its effect on seroma formation should be illuminated with the future studies. We need to apply different doses of phenytoin and change time of usage.

## Competing interest

The authors declare that they have no competing interests.

## Authors’ contributions

ME, FT, MK, SG conceived of the study and participated in its design and coordination. LK, MOG, HU made substantial contributions to data acquisation and conception of manuscript and drafted and designed the manuscript. All authors read and approved the final manuscript.

## Pre-publication history

The pre-publication history for this paper can be accessed here:

http://www.biomedcentral.com/1471-2482/12/25/prepub
